# Spatial and temporal analysis of dengue infections in Queensland, Australia: Recent trend and perspectives

**DOI:** 10.1371/journal.pone.0220134

**Published:** 2019-07-22

**Authors:** Rokeya Akter, Suchithra Naish, Michelle Gatton, Hilary Bambrick, Wenbiao Hu, Shilu Tong

**Affiliations:** 1 School of Public Health and Social Work, Institute of Health & Biomedical Innovation, Queensland University of Technology, Brisbane, Queensland, Australia; 2 School of Health, Medical and Applied Sciences, Central Queensland University, Queensland, Australia; 3 Shanghai Children's Medical Centre, Shanghai Jiao Tong University, Shanghai, China; 4 School of Public Health, Anhui Medical University, Hefei, China; Fundacao Oswaldo Cruz Instituto Rene Rachou, BRAZIL

## Abstract

Dengue is a public health concern in northern Queensland, Australia. This study aimed to explore spatial and temporal characteristics of dengue cases in Queensland, and to identify high-risk areas after a 2009 dengue outbreak at fine spatial scale and thereby help in planning resource allocation for dengue control measures. Notifications of dengue cases for Queensland at Statistical Local Area (SLA) level were obtained from Queensland Health for the period 2010 to 2015. Spatial and temporal analysis was performed, including plotting of seasonal distribution and decomposition of cases, using regression models and creating choropleth maps of cumulative incidence. Both the space-time scan statistic (SaTScan) and Geographical Information System (GIS) were used to identify and visualise the space-time clusters of dengue cases at SLA level. A total of 1,773 dengue cases with 632 (35.65%) autochthonous cases and 1,141 (64.35%) overseas acquired cases were satisfied for the analysis in Queensland during the study period. Both autochthonous and overseas acquired cases occurred more frequently in autumn and showed a geographically expanding trend over the study period. The most likely cluster of autochthonous cases (Relative Risk, RR = 54.52, p<0.001) contained 50 SLAs in the north-east region of the state around Cairns occurred during 2013–2015. A cluster of overseas cases (RR of 60.81, p<0.001) occurred in a suburb of Brisbane during 2012 to 2013. These results show a clear spatiotemporal trend of recent dengue cases in Queensland, providing evidence in directing future investigations on risk factors of this disease and effective interventions in the high-risk areas.

## Introduction

Dengue, a widespread acute infectious disease caused by four different serotypes of dengue virus transmitted by two types of mosquitoes; *Aedes aegypti* (primary vector) and *Aedes albopictus* [1], has emerged as a significant public health problem in tropical and subtropical regions of the world [2]. Globally, dengue incidence has increased 30 fold in the past 50 years [2] with 390 million cases reported annually [3].

In Australia, dengue re-emerged in northern Queensland during 1981–1982 [4]. However, notified cases were first reported to the National Notifiable Disease Surveillance System (NNDSS) during 1992–1993 [5]. Since then, dengue outbreaks with autochthonous cases have been regularly reported in Queensland [6]. To prevent or minimise the challenge imposed by dengue outbreaks throughout Queensland, Queensland Health has implemented an improved surveillance system, while local government conducts mosquito control activities include the use of traps, briquettes, insect growth regulators and bio-controls using *Wolbachia*. However, autochthonous cases are still occurring in Queensland [7, 8].

In Queensland, dengue is not presently endemic. Rather, autochthonous transmission of dengue is initiated when local *Aedes* mosquitoes bite infectious returned travellers [7, 9–12]. In Australia, even though both autochthonous and overseas acquired dengue cases occur, previous research that considered both autochthonous and overseas acquired cases [13–17] mostly investigated factors responsible for dengue transmission, with little consideration of spatial expansion over time and cluster identification. The two studies that did consider spatio-temporal aspects did so at the national [14] and Local Government level [13]. However, the data is now more than one decade old [16].

Since 2005 Queensland has experienced an increased number of overseas travels and visitor arrivals [18, 19] from dengue endemic countries, the arrival of *Aedes albopictus* in the Torres Strait Islands [20] and an increased number of *Aedes* vector incursions in Cairns and Brisbane [20]. It is also projected that the spatial range of dengue vectors will expand into south and west of the country [21], even all over Australia [22] under adaptive measures of inevitable climate change. Thus, it is necessary to further investigate the spatio-temporal trend of both overseas acquired and autochthonous cases at fine spatial scale taking special consideration of recent outbreaks for prioritising resource allocation and dengue control management. Besides, Eliminate Dengue with the support of World Mosquito Programme (WMP) (http://www.eliminatedengue.com/au) started Wolbachia field trial in different areas of Cairns and Townville areas. Under this mosquito control programme whether the spatial and temporal patterns have been changed needs to be investigated.

During recent years, with the rapid development of Geographic Information Systems (GIS), methods of spatial analysis have been increasingly applied to infectious diseases, especially vector-borne diseases [14, 23–25]. Identification of spatial high-risk areas at fine spatial level can help guide local health departments to form public health strategies, resource allocation, initiate early preventive measures and conduct enhanced surveillance, thereby reducing the risk of epidemics.

Considering the lack of research focusing on recent dengue outbreaks in Queensland, and the lack of analysis at fine spatial scale, this study aimed to explore the spatial and temporal trends of both autochthonous and overseas acquired dengue cases during 2010–2015 in Queensland to identify high-risk areas at small geographical scale and thereby help in planning resource allocation for dengue prevention and intervention. Further, this study will help to understand the effect of the release of Wolbachia as a vector control strategy in Queensland.

## Materials and methods

### Study area

Queensland, the second largest state of Australia, occupies 25% of Australia's continent. Population wise it is third largest state with the total population of 4.8 million. It lies between latitudes 10–28° S and longitudes 138–153° E. Brisbane is the capital city of Queensland which is flanked by two most popular surfing beaches named Gold Coasts and Sunshine Coasts. There are 475 Statistical Local Areas (SLA) in Queensland. Climate shows significant variation across the state. Low rainfall and hot summers are common in the inland west. Wet and dry seasons occur in the far north, and coastal strip is characterised by the warm temperate conditions. Four distinct seasons such as summer (December-February), autumn (March-May), winter (June-August) and spring (September-November) are prominent in a year.

### Dengue data

In Australia, dengue is a nationally notifiable disease [5]. All cases are required under the Public Health Act 2005 to be notified to health departments within each state and territory where there is a laboratory confirmation of infection by any one of several different methods including virus isolation, nucleic acid testing, detection of dengue non-structural protein 1 (NS1) antigen and dengue virus-specific IgG seroconversion. Data on dengue notification dates, number of cases, place of residence of the notified cases at Statistical Local Area (SLA) level, and source of infections (either autochthonous or overseas acquired) were obtained from Queensland Health. A patient with recent travel history to a dengue endemic country was considered as an overseas acquired case whereas no travel history was assumed to be an autochthonous case. Both autochthonous and overseas acquired dengue cases were obtained for the period January 2010 to December 2015. This study period was selected to avoid the large epidemic outbreaks in 2003 and 2009 as major public health interventions were implemented at those times. Besides, in our previous research we explored the spatiotemporal patterns of dengue fever for long term data (1993–2012) [12], therefore, in this study we aim to check if the spatial and temporal patterns have been changed after Wolbachia was used on field trial to reduce dengue transmission. Population estimates for the same period were obtained from the Australian Bureau of Statistics.

Of the total cases, 97 were not included in the analysis due to ambiguity of their origin (10 cases) and failure of geocoding due to insufficient or unclear information (11 autochthonous cases and 76 overseas acquired cases).

### Data analysis

Time series seasonal decomposition analysis was performed to explore the impact of seasonality on autochthonous and overseas acquired cases. A seasonal decomposition method was used to break down monthly time series data into four components: original time series data, trend, seasonality and random effect or error component. Linear regression models were also developed to assess the linear trend of annual number of totally affected SLAs and newly affected SLAs for both autochthonous and overseas acquired cases. SLAs with dengue cases occurring for the first time since 2010 were defined as newly affected SLAs in the corresponding year whereas SLAs without any occurrence during study period were regarded as non-affected areas.

### Geographic distribution of dengue incidence

The annual incidence of dengue was calculated for each SLA by dividing the number of annual cases in each SLA by the corresponding SLA population, and multiplying by 100,000. The cumulative incidence of dengue cases by SLA was then calculated and mapped to present the geographic distribution of the occurrence of dengue cases. To assess spatial and temporal patterns of dengue cases at the SLA level from 2010 to 2015, we plotted the newly affected and non-affected SLAs by year. Two additional categories were also added such as previous but not currently affected (occurrence of dengue before calendar year but not in calendar year), and previously and currently affected SLAs (occurrence of dengue both in calendar year and before calendar year) to show temporal variation of the cases over time. ArcMap (version 10.2, 2013) was used to visualise the spatio-temporal patterns or geographic distribution of dengue cases.

### Space-time cluster analysis

The space-time scan statistic (SaT Scan) was applied to autochthonous and overseas acquired dengue cases to test whether the cases were distributed randomly over space and time and to locate space-time clusters and determine their statistical significance. SaT Scan (version 9.4.2) was employed to conduct a retrospective space and time scan assuming cases were Poisson-distributed in each location. The spatial scan statistic is a maximum likelihood ratio test statistic used to find the maximum likelihood ratio while sweeping over all zones bounded by a window with variable sizes and shapes [26]. For each zone, the likelihood ratio is computed by counting the observed number of cases inside and outside that zone. The zone that maximizes the likelihood ratio defines the most likely cluster. Other windows for which the likelihood value was statistically significant will be defined as secondary clusters ranked according to their likelihood ratio test statistic. The cluster statistical significance was investigated with a log likelihood ratio test using the number of Monte Carlo replication sets under the null hypothesis of random distribution. In this study, only the most likely cluster and the secondary clusters are reported if the p-value of the clusters is below 0.05. In this study, SLA in Queensland was used as a spatial unit; there were 475 SLAs in Queensland during the study period. The time unit was a calendar year. In the SaT Scan software, a default maximum spatial cluster size of 50% of the whole population was used to detect the large clusters that tend to have a small relative risk but a high statistical significance. The maximum temporal cluster size was 50% of the study period. ArcMap (version 10.2, 2013) was used to visualize the significant clusters.

## Ethical considerations

The study was approved by the Human Research Ethics Committee, Queensland Health Data Custodian under the Public Health Act, 2005 followed by Research Ethics Unit, Queensland University of Technology (approval number: 1500001029).

## Results

### Descriptive analysis

**Fig 1:**
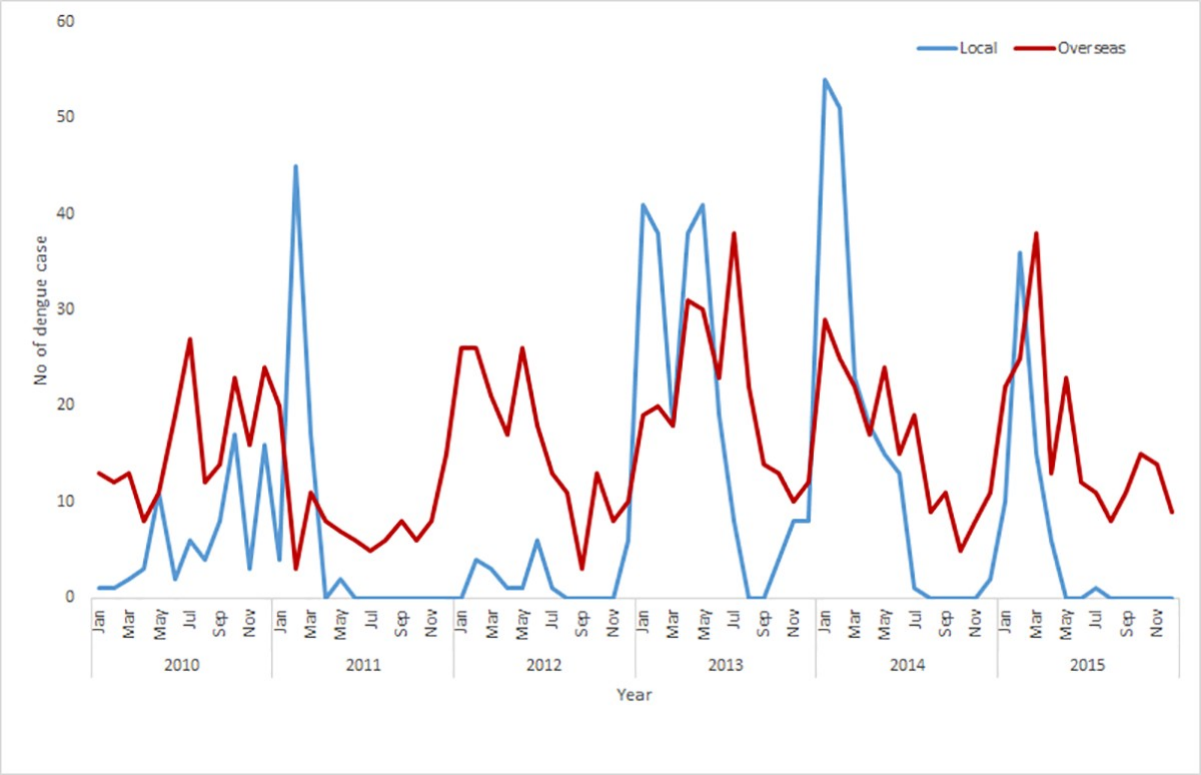
Time series plot of autochthonous and overseas acquired dengue cases during 2010–2015 in Queensland, Australia.

During 2010–2015, a total of 1,773 dengue cases with 632 (35.65%) autochthonous cases and 1,141 (64.35%) overseas acquired cases were satisfied for the analysis in Queensland, Australia. Annual variation in the number of autochthonous cases was striking, with the highest peak occurring in 2013 (224 cases) and the smallest in 2012 (only 22 cases) (Table 1). A total of 10 SLAs (2 for autochthonous cases and 8 for overseas acquired cases) had dengue cases in each of the six years from 2010 to 2015, and 29 SLAs had cases acquired from overseas in five of those years. Fig 1 depicts the monthly distribution of both autochthonous and overseas acquired cases during 2010–2015 with the highest peak of autochthonous cases mostly in November to April and overseas acquired cases in September to April.

**Table 1:** Descriptive statistics of dengue cases in Queensland, Australia during 2010–2015.

Parameter	2010	2011	2012	2013	2014	2015	Total
**All cases**	267	172	213	475	375	271	1773
**Autochthonous case (% of all cases in year)**	74 (27.72)	68 (39.53)	22 (1.33)	224 (47.16)	176 (46.93)	68 (25.09)	632 (35.65)
**Overseas acquired case (% of all cases in year)**	193 (72.28)	104 (60.47)	191 (89.67)	251 (52.84)	199 (53.07)	203 (74.91)	1141 (64.35)
**No. of SLAs with Autochthonous case (Newly affected SLAs)**	17*	8 (3)	5 (1)	23 (10)	21 (8)	11 (3)	-
**No. of SLAs with Overseas acquired case (Newly affected SLAs)**	118*	83 (34)	113 (47)	152 (50)	140 (31)	125 (20)	-

* As the study period starts from 2010, number of newly affected SLAs in this year were not possible to count

### Seasonality of autochthonous and overseas acquired dengue cases

**Fig 2:**
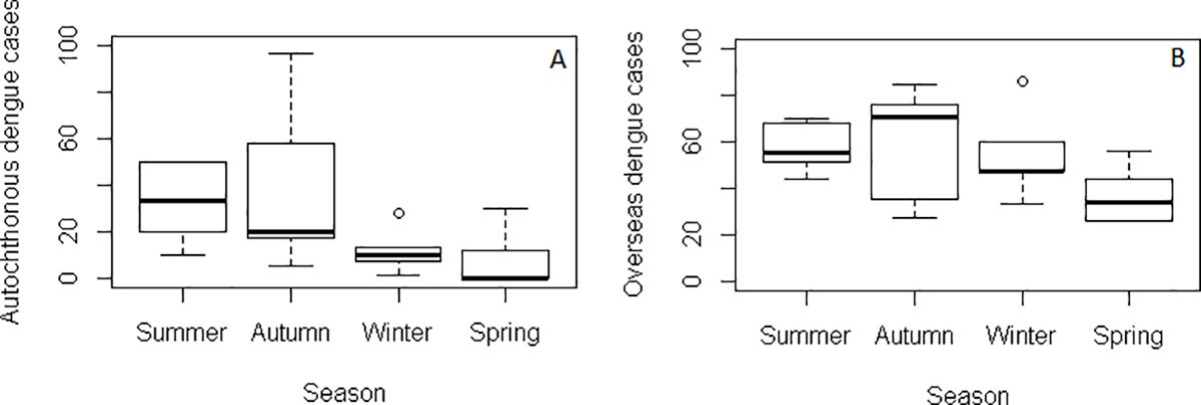
Seasonal distribution of autochthonous (A) and overseas acquired cases (B). Summer, December-February; autumn, March-May; winter, June-August; spring, September-November.

**Fig 3:**
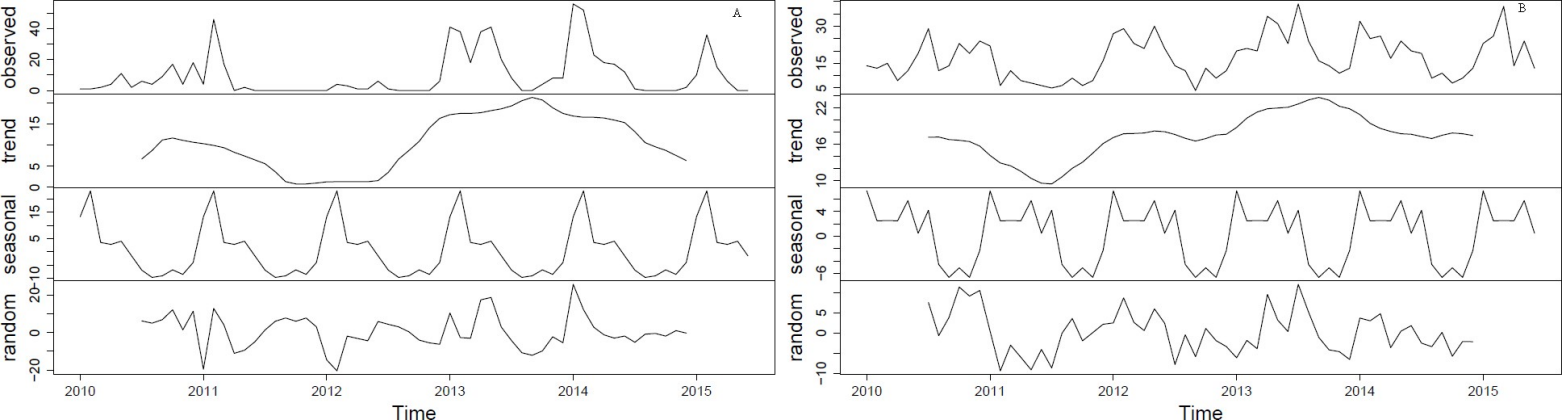
Seasonal decomposition of autochthonous (A) and overseas acquired cases (B).

The occurrence of both autochthonous and overseas acquired cases showed distinct seasonal characteristics. Both autochthonous and overseas acquired cases were relatively rare in winter and spring, with a slightly higher number in summer, and a peak in the autumn (Fig 2). However, the seasonal decomposition analysis showed that there is an increasing trend of dengue infections with distinct seasonality for both autochthonous and overseas acquired cases. The seasonality in both autochthonous and overseas acquired cases is fixed and constant between years and always followed the same pattern throughout the study period (Fig 3).

### Spatio-temporal trend of dengue affected areas

**Fig 4:**
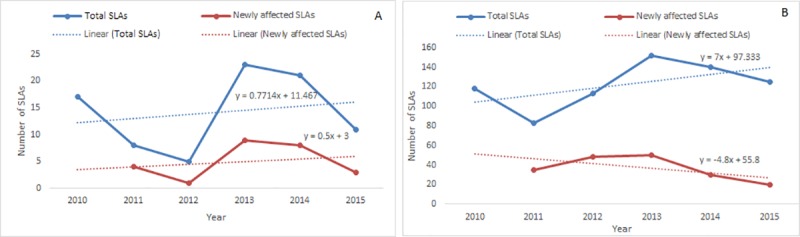
Trends of number of totally affected SLAs and newly affected SLAs for autochthonous (A) and overseas acquired cases (B).

Regression models showed that the number of total and newly affected SLAs for autochthonous infections followed an increasing trend. The annual number of total affected SLAs for autochthonous infections increased by 0.77 fold (p = 0.71) whereas it is 7 fold (p = 0.26) in overseas acquired cases (Fig 4). The annual number of newly affected SLAs for autochthonous cases increased by 0.5 fold (p = 0.70). However, these results were not statistically significant which might be due to short length of the study period. Even though per year newly affected SLAs for overseas acquired cases showed decreasing trend, however, each year occurrence of a single affected new area indicates the sign of expansion of dengue affected areas. Further, this could be due to the lack of information of newly affected SLAs in 2010.

### Geographic distribution of dengue incidence

**Fig 5:**
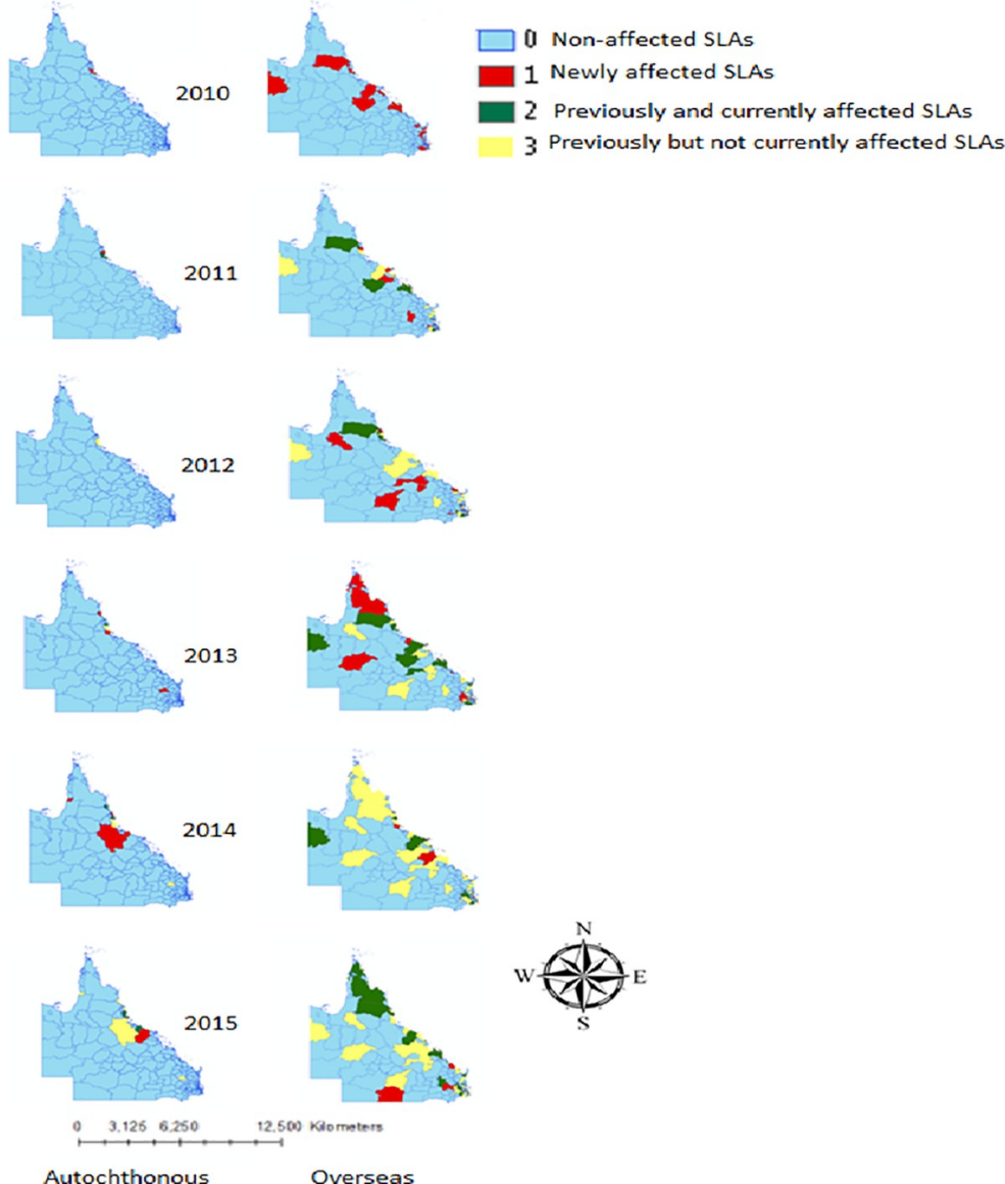
Spatio-temporal distribution of autochthonous and overseas acquired cases in Queensland, Australia during 2010–2015. SLAs are categorised as “Non-affected” meaning no occurrence of dengue during study period; “Newly affected” meaning occurrence of dengue for the first time since 2010; “previously and currently affected” meaning occurrence of dengue both in calendar year and before calendar year; “Previously but not currently affected” meaning occurrence of dengue not in calendar year but before calendar year.

Newly affected SLAs (with both autochthonous and overseas acquired cases) were observed near Cairns each year (Fig 5). Although overseas acquired cases were observed all over Queensland, autochthonous cases were only confined to Cairns and Cassowary Coast and Townsville areas.

**Fig 6:**
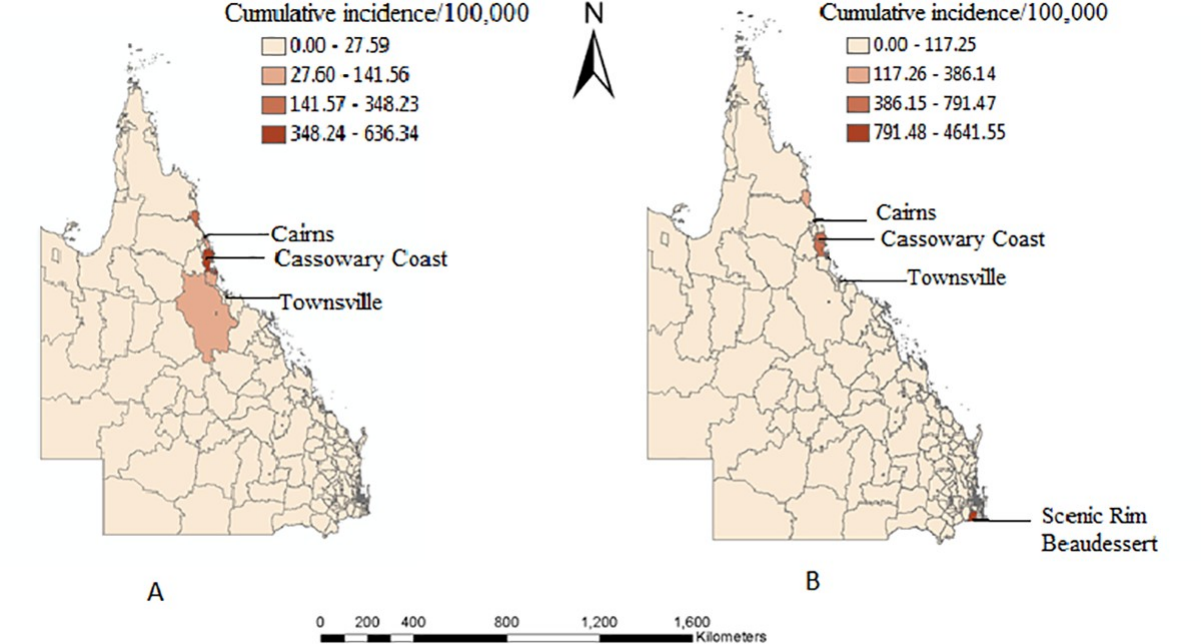
Cumulative incidence of autochthonous (A) and overseas acquired cases (B) during 2010–2015.

Furthermore, our results showed that the highest number of dengue cases was in Cairns, Cassowary Coast and Townsville areas with a cumulative incidence rate of 348/100,000 for autochthonous and 790/100,000 for overseas acquired dengue occurrence (Fig 6).

### Space-time cluster analysis

Table 2 shows the results of the spatio-temporal cluster analysis, stratified by autochthonous and overseas acquired cases. Using a maximum spatial cluster size of ≤50% of the total population, only one statistically significant cluster of autochthonous cases was detected, which included 50 SLAs around the Cassowary coast (RR = 54.52, radius = 225.83km, p<0.001) during 2013–2015.

**Table 2:** Clusters of dengue fever by autochthonous and overseas acquired cases in Queensland, Australia, 2010–2015.

Spatio temporal	Cluster	Radius (Km)	Start date	End date	No. of SLAs	LLR	O (n)	E(n)	P value	RR
**Autochthonous**	1	225.830	2013/1/1	2015/12/31	50	1047.14	460	29.55	0.001	54.52
**Overseas**	1	0	2012/1/1	2013/12/31	1	31.19	10	0.17	0.001	60.81
	2	0	2012/1/1	2014/12/31	1	28.56	6	0.02	0.001	317.29
	3	36.72	2012/1/1	2014/12/31	9	25.42	59	20.05	0.001	3.05
	4	26.27	2012/1/1	2014/12/31	26	24.09	92	41.37	0.001	2.33
	5	6.36	2013/1/1	2015/12/31	39	18.21	76	35.41	0.001	2.23
	6	4.70	2013/1/1	2015/12/31	2	17.18	17	2.68	0.001	6.43

RR, Relative Risk; LLR, Log Likelihood Ratio; O, observed cases; E, expected cases; p value, significant at 5% confidence interval.

**Fig 7:**
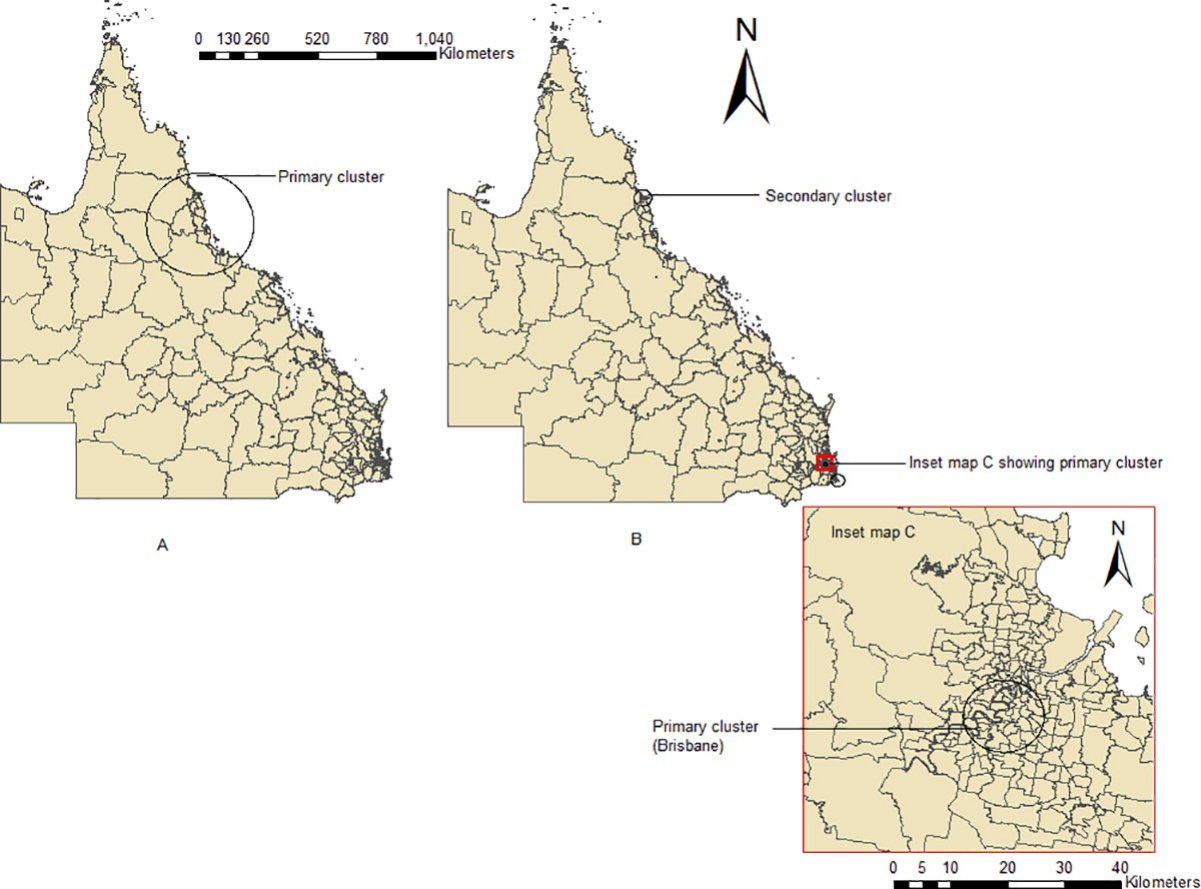
Space-time clusters of autochthonous (A) and overseas acquired cases (B).

For the overseas acquired cases, using the same spatial cluster size, 6 statistically significant clusters were identified with the most likely cluster in Herston, Brisbane, during 2012–2013 with a RR of 60.81 (p<0.001 comprising only one SLA (n = 1)) and the secondary clusters occurred in Cairns (R)—Central Suburbs (n = 9), Scenic Rim (R)–Beaudesert (n = 1), Bilinga-Tugun (n = 26), Yeronga (n = 39) and Noosa-Noosaville (n = 2). All the primary clusters for both autochthonous and overseas acquired cases are represented in Fig 7.

## Discussion

This study presents the spatio-temporal trend of dengue cases stratified by their origin at SLA level in Queensland, Australia. During the study period the most severe outbreaks were during 2013 and 2014 in northern tropical Queensland. Several mosquito control activities such as residual and chemical insecticides spray were administered by the Dengue Action Response Team (DART) in the outbreak region [27]. DART use Biogents Sentinel (BG) traps, Briquets, Insect growth regulators (IGR), and Gravid Aedes Traps (GATs) to control and monitor adult *A*. *aegypti* numbers in high risk areas in north Queensland. The DART also carries out the *A*. *albopictus* control program in the Torres Strait Islands [20]. The Department of Agriculture and Water Resources also work on vector surveillance activities at first ports of entry into Australia. Even with this mosquito control activity in place, there was an increase in the number of dengue cases during the study period. This could be due to delay in notification, local climate variability enhancing mosquito growth and development as well as other socio-ecological factors such as overseas arrivals, movement of people, water tank installation, human behaviour and vegetation types.

Since 2015, Queensland Health, in collaboration with local government, has developed the Queensland Dengue Management Plan 2015–2020 (DMP) which focuses on key areas of dengue management that are international best practice such as vector surveillance and control, outbreak management, case surveillance and public health management, and public awareness and community engagement. Further, Eliminate Dengue is conducting open field trial a novel bio-control strategy that will reduce the ability of *A*. *Aegypti* to transmit dengue virus. The approach is centred on releasing *A*. *aegypti* infected with selected strains of the bacterial endosymbiont *Wolbachia*. Field release trials in north Queensland have demonstrated that *Wolbachia* can be rapidly driven to fixation in populations by a process of selective inheritance, known as cytoplasmic incompatibility. This process impaired the mosquito to transmits dengue virus with ultimate reduction of dengue cases. This might be the reason for low number of local dengue cases in the last 2–3 years.

Seasonal decomposition analysis showed that both autochthonous and overseas acquired dengue cases were highest in autumn (March-May), followed by summer (December-February). Our finding is consistent with previous studies that have reported a strong seasonal pattern of dengue in north Queensland [28–30].

In Australia, summer (December to February) is the season with the highest rainfall, with cyclones affecting northern areas. Besides this, Christmas and extended summer school holidays occur during this period. Hence, it is a popular time for Australians to visit overseas countries where dengue is endemic and where they may have acquired dengue virus. Asia, predominantly Southeast—Asia has traditionally been reported as the major source of virus importation into Australia [9, 31]. The seasonal pattern of epidemics in these countries and the seasonality of travel may be the possible reasons for overseas cases presenting a seasonal pattern.

Our findings also indicated that newly affected areas appear to have been expanding in Queensland over recent years, which indicates potentially increasing risk for unaffected areas in and around Cairns (Fig 5). Dengue outbreaks in 2019 in central Queensland are an example of southward movement of dengue distribution (https://www.health.qld.gov.au/clinical-practice/guidelines-procedures/diseases-infection/diseases/mosquito-borne/dengue/dengue-outbreaks). In 2013 local dengue transmission was observed in South Queensland. Two explanations are possible; either dengue infections may have occurred locally, but literature does not support the presence of primary *A*. *aegypti* in this region [32]; this region is classified as a lower risk area. However, the risk of transmission classification for large parts of Queensland is uncertain due to the paucity of vector surveillance data [7]. Alternatively, this could be explained by movement of the infected person from place of infection (north Queensland) to residential area (i.e., the patient may be infected in north Queensland via domestic travelling). The second explanation is supported by the scientific evidence which reported that human movement is responsible for dispersal of dengue virus over space [33].

Space-time cluster analysis demonstrated that autochthonous cases were clustered in 50 SLAs in the Cassowary Coast-Cardwall region, including Cairns. Most of the clusters occurred during 2012 to 2015. Overall distribution of the cases may be underpinned by travel and improved transport system which facilitates the distribution of mosquito as well as human hosts. Socio-demographic factors may also contribute to the cycles of dengue transmission [34]. The increasing trend of dengue cases and socio-ecological factors in Queensland may be linked in the geographical expansion of dengue cases by providing suitable habitat for *Aedes* mosquito [18], and thus, contribute to the formation of autochthonous clusters of dengue in this region. This could also be due to the reopening of the Cairns international air service, enabling overseas visitors to visit Cairns and nearby regions directly and thereby possibly increasing the chances of autochthonous and overseas acquired cases. Furthermore, increased overseas commercial investment, export of labour and foreign tourism might have led to increased activity in international exchanges in this region, thus, increasing the risk of imported dengue cases from endemic areas. Most of the overseas acquired cases in Queensland have been imported from Southeast Asian countries, especially Indonesia, Thailand, Philippines, Singapore and Papua New Guinea [9, 31]. A single study has also reported that dengue cases from Southeast Asian countries have triggered autochthonous outbreaks in northern Queensland [16]. In this study, the reason for cluster formation has not been explored. Therefore, future research focusing on identification of risk factors of dengue might explain why dengue is confined to north Queensland whereas preferred temperatures (>16°C) [35] of *Aedes* mosquito is almost present all over Queensland.

The most likely cluster of overseas acquired cases occurred in Herston, Brisbane. As there is no *Aedes* mosquito in the Brisbane area, it cannot initiate any autochthonous transmission. However, an entomological household survey in Brisbane found that approximately 26% of all premises surveyed harboured *A*. *notoscriptus* [36]. It is interesting to note that both *A*. *aegypti* and *A*. *notoscriptus* have similar ecologies to *A*. *albopictus* [37], and a study has discussed the overlapping capacities of their habitats [38] as well as the potential displacement of native species by the more aggressive *A*. *albopictus* [20]. In recent times, *A*. *albopictus* has been established in Torres Strait Islands and caused the 2016 outbreak in that region [20, 39], and also increased the chance of this mosquito becoming established in mainland Australia, especially Cairns which is 800 km away from Torres Strait Island [20]. Further, several incursions of *A*. *albopictus* also occurred in different ports such as Cairns, Brisbane and Townsville [20]. Given the presence of imported dengue cases as well as several incursions of *A*. *albopictus* in this region, future local transmission is likely. An economic cost analysis of severity of dengue posed by establishment of *A*. *albopictus* in Brisbane found mosquito control program would be cost-effective rather than ignoring the possibility of establishment [39] suggesting that household level surveillance could be a feasible approach.

Our results seem to reflect similar patterns observed in the previous study [13], albeit at a fine spatial scale. Hu et al. (2012) showed high-incidence LGA (Local Government Areas) clusters for locally acquired infections were in north Queensland where as high-incidence clusters for overseas acquired cases were in north and south-east Queensland. Therefore, the patterns seem consistent over time and method of analysis.

The strengths of the study are as follows: most of the previous spatio-temporal studies of dengue in Australia [11, 12] as well as other countries such as in Bangladesh [40], Thailand [41], Sri Lanka [42] largely focused on autochthonous cases. However, this study has included both autochthonous and overseas acquired cases at fine spatial scale with special focus on recent outbreaks. Thus, this study has provided more specific information on clusters for local health departments facing the different risks of autochthonous and overseas acquired dengue cases. Secondly, we considered three different indices (previously but not currently affected areas, both previously and currently affected areas, and newly affected areas) to show the geographic expansion of dengue infections at fine spatial scale. This will allow for the easy identification of geographic expansion of disease activity for resource allocation and mosquito control program.

Potential limitations to this study could firstly be underreporting and misreporting of both autochthonous and overseas acquired cases due to asymptomatic infection. Secondly, the exact place/location (i.e., residential address) where dengue cases were notified may vary from those where they were infected/ acquired, particularly during holiday periods. Use of relatively short time frame for the spatio-temporal distribution may subject to bias.

In summary, this study demonstrates the spatial and temporal expansion of both autochthonous and overseas acquired cases. This expansion might be due to recent changes of social, ecological and demographic factors, for example, human behavioural change, movement of population, overseas arrivals, water tank installation as well as environmental changes (such as climate change and urbanization) in Queensland. Socio-demographic and ecological factors provide natural habitat for mosquito, and increased travel and transport increase the chance of dengue virus importation. Therefore, all health professionals need to be aware of dengue risk in returning travellers, particularly from dengue endemic areas. It is necessary to implement mosquito control measures in the areas with the highest percentage of returned travellers. Local health departments could take early preventive measures, conduct enhanced surveillance and prioritise resource allocation in the high-risk areas to reduce the risk of epidemics. Additional implication of the study includes future investigation of identifying risk factors and effective interventions in the high-risk areas for the control of dengue and other vector-borne diseases.
